# Acute Elevated Glucose Promotes Abnormal Action Potential-Induced Ca^2+^ Transients in Cultured Skeletal Muscle Fibers

**DOI:** 10.1155/2017/1509048

**Published:** 2017-08-01

**Authors:** Erick O. Hernández-Ochoa, Quinton Banks, Martin F. Schneider

**Affiliations:** Department of Biochemistry and Molecular Biology, University of Maryland School of Medicine, Baltimore, MD 21201, USA

## Abstract

A common comorbidity of diabetes is skeletal muscle dysfunction, which leads to compromised physical function. Previous studies of diabetes in skeletal muscle have shown alterations in excitation-contraction coupling (ECC)—the sequential link between action potentials (AP), intracellular Ca^2+^ release, and the contractile machinery. Yet, little is known about the impact of acute elevated glucose on the temporal properties of AP-induced Ca^2+^ transients and ionic underlying mechanisms that lead to muscle dysfunction. Here, we used high-speed confocal Ca^2+^ imaging to investigate the temporal properties of AP-induced Ca^2+^ transients, an intermediate step of ECC, using an acute in cellulo model of uncontrolled hyperglycemia (25 mM, 48 h.). Control and elevated glucose-exposed muscle fibers cultured for five days displayed four distinct patterns of AP-induced Ca^2+^ transients (phasic, biphasic, phasic-delayed, and phasic-slow decay); most control muscle fibers show phasic AP-induced Ca^2+^ transients, while most fibers exposed to elevated D-glucose displayed biphasic Ca^2+^ transients upon single field stimulation. We hypothesize that these changes in the temporal profile of the AP-induced Ca^2+^ transients are due to changes in the intrinsic excitable properties of the muscle fibers. We propose that these changes accompany early stages of diabetic myopathy.

## 1. Introduction

Diabetes mellitus (DM), a complex metabolic syndrome, is due to the inability of the pancreas to produce and/or secrete insulin, referred as insulin deficiency or improper insulin signal transduction by tissues like hepatic, fat, and skeletal muscle, known as insulin resistance. In either insulin deficiency or resistance, the cells are unable to adequately metabolize the glucose, leading to hyperglycemia, the hallmark of the disease. Late complications of diabetes affect both the quality and quantity of life, resulting in major health costs [1]. The disease progression of both type 1 (T1D) and type 2 (T2D) diabetes are different, yet the clinical manifestations and complications are often similar [1]. During episodes of hyperglycemia, glucose levels reach abnormal elevated values ranging from 120 to 1200 mg/dL [2–4]. In addition to the change in glucose concentration, hyperglycemia is accompanied by significant changes in plasma osmolarity [2, 4, 5]. Individuals affected by long-term T2D repeatedly present modest but significant changes in glucose concentration and osmolarity, while patients with acute uncontrolled hyperglycemia (i.e., T1D) can exhibit even larger changes in osmolarity [2–5]. Consequently, it is anticipated that harmful effects of hyperglycemia and/or hyperglycemic-induced osmotic stress contributes to the progression of diabetic complications and comorbidities.

A common comorbidity of both T1D and T2D is sarcopenia and dynapenia—the loss of muscle mass and strength, respectively, and is termed diabetic myopathy [6, 7]. The adequate function of skeletal muscle is fundamental for body movement and glucose metabolism [8–10], and the development of diabetic myopathy, an understudied and commonly overlooked condition, is believed to worsen the metabolic status of the individual already affected with concurrent diabetic complications. Comprehensive studies involving large numbers of patients with chronic T2D have shown increased sarcopenia and dynapenia when contrasted to healthy individuals [11, 12]. Fatigue and weakness are also common findings in patients with acute episodes of hyperglycemia, particularly in patients with T2D [13]. There are numerous studies related to fatigue, sarcopenia, and dynapenia [14–18]; nevertheless, the precise cellular events linked with these muscular conditions in individuals afflicted by diabetes remain unidentified.

While previous studies have investigated the link between changes in skeletal muscle function and muscle mass [1, 7, 10–12, 14, 17–23], Ca^2+^ homeostasis and signaling in different models of long-term diabetes mellitus [24–27], few have examined the impact of relatively acute elevated glucose on action potential- (AP-) induced Ca^2+^ transients. Direct acute effects of hyperglycemia could have implications for the skeletal muscle myopathy seen in diabetes, especially in patients with poor glycemic control. In particular, studies of experimental diabetes in skeletal muscle have shown alterations in the excitation-contraction coupling (ECC)—a coordinated chain of cellular events that link membrane AP, intracellular Ca^2+^ release, and contractile machinery [28, 29]. We previously reported that muscle fibers exposed to elevated glucose display increased AP-evoked Ca^2+^ signals produced by single brief electric stimulation [25]. Yet, little is known about the consequences of acute elevated glucose on the temporal properties of AP-induced Ca^2+^ transients and the underlying ionic mechanisms that lead to muscle dysfunction. Here, we used ultra-high-speed confocal Ca^2+^ imaging to investigate the temporal properties of AP-induced Ca^2+^ transients, an intermediate step of ECC, using a cellular model of acute hyperglycemia. Our results reveal that elevated glucose-exposed fibers predominantly display abnormal AP-induced Ca^2+^ transients.

## 2. Methods

### 2.1. FDB Skeletal Muscle Fibers Culture

Studies were performed on skeletal muscle fibers enzymatically isolated from the *flexor digitorum brevis* (FDB) muscles of 4- to 5-week-old C57BL/6J mice as previously described [30–33]. Mice were euthanized by CO_2_ exposure followed by cervical dislocation using protocols approved by the University of Maryland Institutional Animal Care and Use Committee. FDB skeletal muscle fibers were isolated, dissociated, and cultured in a humidified incubator at 37°C (5% CO_2_) as previously described [30–33]. FDB muscles were dissected and maintained in minimum essential medium (MEM, Life Technologies, Carlsbad, CA, catalog number 11095080) and 2 mg/mL collagenase type I (Sigma-Aldrich, St. Louis, MO, catalog number C-0130) for 3 h. at 37 °C. Muscle fibers were plated on glass-bottomed culture dishes (Matek Inc., Ashland, MA, catalog number P35G-1.0-14-C) coated with laminin (Life Technologies, Carlsbad, CA, catalog number 23017015). After plating, cultures were incubated in MEM, containing 5.56 mM D-glucose, supplemented with 10% fetal bovine serum (FBS, Life Technologies, Carlsbad, CA, catalog number 10100139) and 50 *μ*g·ml^−1^ gentamicin (Life Technologies, Carlsbad, CA, catalog number 15710064). This medium was used as a control isotonic condition (288 mOsm/kg). Two hours after plating, cultures were treated with cytosine *β*-D-arabinofuranoside (ara-C; Sigma-Aldrich, St. Louis, MO, catalog number C-1768; 10 *μ*M for 24 h.) to reduce proliferating cells and to minimize fiber dedifferentiation [30, 33]. For fibers challenged with elevated extracellular glucose media, either D- or L-glucose (25 mM; 48 h.) was added to the control isotonic medium. Over an isotonic reference of 288 mOsm/kg, the addition of 25 mM D-glucose increased the osmolality to 313 mOsm/kg. Osmolarity of the culture media was measured in a Vapro-5520 Osmometer (Wescor Inc., Logan, UT). Here, muscle fiber cultures were 5 days old when used for acute experiments.

### 2.2. Ca^2+^ Imaging

**Figure 1:**
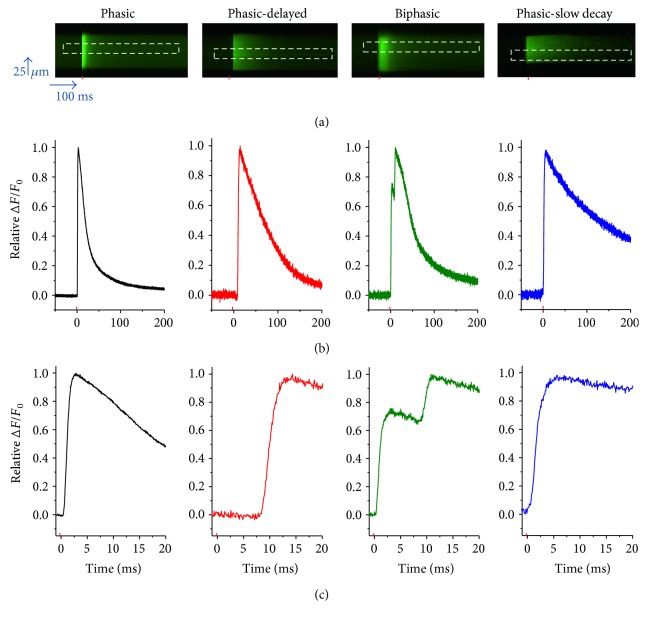
Muscle fibers cultured for 5 days exhibit multiple patterns of action potential (AP)-induced Ca^2+^ transients: control conditions. (a) Representative confocal line-scan images of AP-induced Ca^2+^ transients in 5-day-old cultured muscle fibers maintained in control medium. Note that four different patterns were identified: phasic, phasic-delayed, biphasic, and phasic-slow-decay. The red mark indicates the time when field electrical stimulus was applied, and the dashed rectangle illustrates the region of interest used to measure the time course of the Ca^2+^ transient. (b) Time course of the AP-induced Ca^2+^ transients shown in (a). (c) Zoomed-in versions of AP-induced Ca^2+^ transients shown in (b).

**Figure 2:**
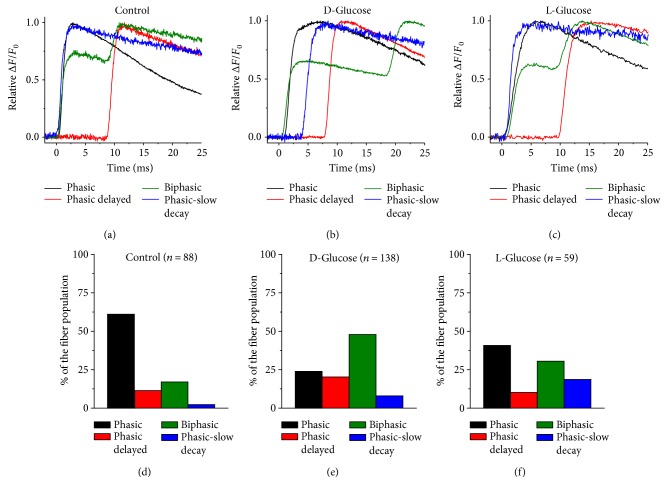
Sustained elevation of extracellular D-glucose modifies the distribution of AP-induced Ca^2+^ transients observed in 5-day-old cultured fibers. Zoomed-in and overlapped version of AP-induced Ca^2+^ transients for control (a), D-glucose (b), and L-glucose (c) challenged fibers. (d–f) Summary of distribution of AP-induced Ca^2+^ transients for fibers exposed to control isotonic medium (d), D-glucose (e), and L-glucose (f). Fibers exposed to D-glucose displayed a significantly larger proportion of biphasic action potential-induced Ca^2+^ transients when compared to control counterparts (*X*^2^, *n* = 286, *p* value < 0.05).

Fluo-4 measurements were carried out on a high-speed confocal system (LSM 5 Live, Carl Zeiss, Jena, DE) as previously described [34, 35]. Muscle fibers were loaded with 1 *μ*M fluo-4 AM (Life Technologies, Carlsbad, CA, catalog number F14201) in L-15 medium (Life Technologies, Carlsbad, CA, catalog number 21083027). The ionic composition of L-15 in mM is 137 NaCl, 5.7 KCl, 1.26 CaCl2, and 1.8 MgCl2, pH 7.4) supplemented with 0.25% *w*/*v* bovine serum albumin (BSA; Sigma-Aldrich, St. Louis, MO, catalog number A-7906) for 1 h. at room temperature. The dishes were rinsed once with L-15 medium for 5 min to remove residual fluo-4 AM. Individual muscle fibers were imaged with a 60×/1.3 NA water-immersion objective lens. Excitation for fluo-4 was provided by the 488 nm line of a 100 mW diode laser, and emitted light was collected at >505 nm. Action potential- (AP-) induced Ca^2+^ transients were triggered using a brief electrical field stimulus. External field stimulation and inclusion and exclusion criteria of the characteristics of muscle fibers used in this study were performed as previously described [25, 36]. Supramaximal field stimulation (1 ms square pulse, 30 V/cm) was produced by a custom pulse generator and applied via two platinum wires positioned perpendicular to the bottom of the dish, ∼5 mm apart, to elicit action potentials. Muscle fibers were centrally positioned relative to the electrodes and to the field of view, at less than about a ±45° angle relative to an imaginary line between the tips of the electrodes, and only fibers exhibiting all or no activation and reproducible responses to field stimulation of alternating polarity were used for the analysis. A variable range of the cultured muscle fibers (3–7%) from the control group or from other groups challenged with elevated glucose did not respond to electrical stimulation of both polarities and were excluded from the analysis. Electrical field stimulation was synchronized relative to the beginning of acquisition. The field stimulus was applied 100 ms after the beginning of the scan sequence, providing control images before stimulation. Confocal line scanning was performed at the ends of the fibers and perpendicular to the long axis of the fibers. These line-scan confocal images were used to calculate the resting steady-state fluorescence level (*F*_0_). The average intensity of fluorescence within selected regions of interest (ROIs; dashed rectangles shown in line-scan images in Figures 1 and 2) within a myofiber was measured with Zeiss LSM Image Examiner (Carl Zeiss, Jena, Germany). The ROIs were located in areas spanning the edge and center of the muscle fiber to monitor Ca^2+^ signals derived from subsarcolemmal and core regions of the fiber, or in regions covering the edges of the fiber to monitor the responses across the fiber width. Images in line-scan (*x*-*t*) mode (frame size: 512 × 10,000 pixels; scan speed: 100 *μ*s/line for 1 s acquisition) were background corrected by subtracting an average value recorded outside the cell. The average *F*_0_ value in each ROI before electrical stimulation was used to scale Ca^2+^ signals in the same ROI as Δ*F*/*F*_0_.

It is important to note that the temporal resolution of the Ca^2+^ transient is exclusively determined not only by the sampling rate but also by the kinetic properties of the dyes. Here, we used fluo-4, a high-affinity dye, instead of low-affinity dyes merely because in our imaging system this dye provides brighter responses than the low-affinity indicators. Based on our calibration data, fluo-4 was at most 40% saturated with Ca^2+^. The length of the fibers used was 400–600 *μ*m and the width was 25–80 *μ*m. No attempts were made to distinguish muscle fiber types or to estimate the actual cytosolic Ca^2+^ concentration. Ca^2+^ imaging experiments were carried out at room temperature, 21–23°C.

### 2.3. Toxins and Channel Blockers

To assess the contribution of different ion channels to the development of the biphasic action potential Ca^2+^ transient, 5-day-old cultured fibers were exposed to either gadolinium (Axxora, San Diego, CA, catalog number 400-023-M500), apamin (Sigma-Aldrich, St. Louis, MO, catalog number A-1289), or Jingzhaotoxin-III (JZTX-III; Alomone Labs, Jerusalem, IL, catalog number STJ-200), blockers of mechanosensitivity, SK channels, and Na_v_1.5 channels, respectively. Ion channel blockade treatment was carried out using semilocal perfusion. The working concentration of the blockers used here was based on maximal blocking effects described in previous reports [37–39]. Fibers with biphasic action potential-induced Ca^2+^ transient were first identified, then the time course of the Ca^2+^ profile was assessed before and 10 minutes after blocker application.

### 2.4. Data Analysis

Images of arbitrarily selected muscle fibers were collected and evaluated blindly, using the same settings and enhancing parameters so that all images could be directly compared. Line-scan images were analyzed using LSM examiner (Carl Zeiss, Jena, DE). AP-induced Ca^2+^ signals and statistical analysis were conducted using Origin Pro 8 (OriginLab Corporation, Northampton, MA, USA) and SPSS for Windows ver. 24.0 (SPSS Inc., Chicago, IL, USA). Summary data were reported as mean ± SD. Normal distribution of data was assessed using the Kolmogorov-Smirnov test. Unpaired two-sample Student's *t*-test was used to test for differences between the means of the interspike interval of AP-induced Ca^2+^ transients from two different samples. For experiments that involved the measurements of AP-induced Ca^2+^ transients before or after the addition of ion channel blockers, the interspike interval was compared before and after the addition of blocker in the same muscle fiber. The statistical significance of interspike interval was analyzed using a paired Student's *t*-test on raw data. Cross-classifications and crosstabs were used to examine the relationship between two categorical variables. To test for significant differences, we compared the proportion in each variable, with condition (control and elevated glucose) as the independent variable and AP-induced Ca^2+^ transient pattern (phasic, biphasic, delayed, and slow decay) as the dependent variable, using Pearson chi-square (*X*^2^). Differences were considered significant when *p* value < 0.05.

## 3. Results

### 3.1. Action Potential-Induced Ca^2+^ Transients in 5-Day-Old Cultured Control Fibers

Freshly dissociated or 1- to 3-day-old cultured FDB muscle fibers retain many properties of in situ fibers, including muscle contractions and Ca^2+^ transients that correlate with the number and frequency of field stimulation used. In response to a single 1 ms field stimulus, they respond with one single action potential, a single Ca^2+^ transient, and a single twitch) [30, 33], even when challenged with acute (1 h.) elevated glucose (see Supplementary Figure 1 available online at https://doi.org/10.1155/2017/1509048). In a previous study, we analyzed Ca^2+^ handling and action potential- (AP-) evoked Ca^2+^ transients in 5-day-old cultured control and high-glucose-exposed FDB fibers using a ratiometric Ca^2+^ dye and low temporal resolution [25]. To gain better time resolution of the Ca^2+^ transients, we next monitored fluo-4 transients during stimulation of FDB fibers using an ultra-high-speed (100 *μ*s/line) confocal microscope in line-scan mode.  Figure 1() illustrates representative *x*-*t* confocal line-scan images (Figure 1(a)) and corresponding fluo-4 Ca^2+^ transients (Figure 1(b)) of four different muscle fibers in control conditions and in response to a supramaximal single field stimulus. Confocal line scanning was performed at one end of the fiber and perpendicular to the long axis of the fiber. Successive vertical lines in each line-scan image reveal the time course of the fluorescence signal before and during repetitive stimulation at 100 *μ*s resolution. At first glance, the line-scan images appear to display a single transient in the different fibers shown in Figure 1(a). However, fluo-4 Ca^2+^ profiles (Figure 1(b)) show the time course of the Ca^2+^ signals in more detail. In order to evaluate and compare the temporal profile of the Ca^2+^ signals elicited by action potentials, Ca^2+^ transients were normalized relative to peak maximum fluorescence. Using this approach, we identified four distinct and predominant AP-evoked Ca^2+^ transient profiles in 5-day-old cultured control fibers: phasic, phasic-delayed, biphasic, and phasic-slow decay. To further appreciate the temporal properties of these different Ca^2+^ signals elicited by field stimulation, the fluo-4 Ca^2+^ transients are shown in a time-expanded version in Figure 1(c). These distinct patterns of Ca^2+^ signals and their distributions in percentages were phasic (67%), biphasic (18%), phasic-delayed (12%), and phasic-slow decay (3%) (see Figure 2()). The rising phase of the Ca^2+^ transient following single stimulation occurred within ~1-2 ms of the applied field stimulus in muscle fibers with phasic responses (Figure 1()). In contrast, muscle fibers with a rising phase starting >3 ms after the start of the stimulation were classified as delayed; the duration of this delay was variable (3–15 ms). Another group of fibers exhibited two summated Ca^2+^ transients in response to a single field stimulus, with the first response within 1-2 ms and the second response delayed as in the phasic-delayed fibers, and were classified as biphasic. Finally, another group of fibers exhibited a slow half-time of decay of >100 ms (not shown on the time scale of Figure 1()), and were classified as phasic slow decay. These results indicate that 5-day-old cultured FDB fibers exhibit a heterogeneous fiber population that responds to single field stimulation different to freshly dissociated or 1-day-old cultured fibers [31, 35].

### 3.2. Action Potential-Induced Ca^2+^ Transients in 5-Day-Old Cultured Muscle Fibers Challenged with Elevated D-Glucose or L-Glucose

In another series of experiments, muscle fibers were challenged with elevated glucose (25 mM). We assessed the properties of AP-induced Ca^2+^ transients in fibers challenged with either D-glucose or L-glucose (Figure 2()) using the same approach applied to 5-day-old cultured control fibers. Fibers exposed to elevated glucose displayed the same patterns of AP-induced Ca^2+^observed in control fibers; however, the distribution of the patterns was different (Figure 2()). In D-glucose exposed fibers (Figures 2(b) and 2()) the distribution was: biphasic (48%), phasic (24%), phasic-delayed (21%), and phasic-slow decay (7%). In L-glucose exposed fibers (Figures 2(c) and 2()) the distribution was: phasic (41%), biphasic (30%), phasic-slow decay (19%), and phasic-delayed (10%), whereas in control the distribution was phasic (67%), biphasic (18%), phasic-delayed (12%), and phasic-slow decay (3%) (Figures 2(a) and 2()). We tested whether elevated glucose-exposed fibers exhibit different distribution of patterns of AP-induced Ca^2+^ transients when compared to control counterparts. The two-sided asymptotic significance of the chi-square statistic was less than 0.05; *X*^2^ (6, *n* = 286) 43.08 *p* = 1.12*E* − 7, implying that elevated glucose-exposed fibers exhibit a different distribution of patterns of AP induced-Ca^2+^ transients when compared to control counterparts. Note that the biphasic pattern was more commonly observed in D-glucose challenged fibers, while in L-glucose exposed fibers the phasic pattern was predominant, as seen in control fibers (Figure 2()). Also, the interspike interval in biphasic D-glucose challenged fibers was significantly longer (17.9 ± 4.2 ms in D-glucose versus 8.3 ± 3.4 ms in control or 9.1 ± 3.8 ms in L-glucose, in *n* = 12 fibers, 3 mice per group; *p* = 0.09, two-sample unpaired Student's *t*-test). The effects of D-glucose on the distribution of the AP-evoked Ca^2+^ transients were distinct to those observed in muscle fibers exposed with the same concentration of metabolically inactive L-glucose (Figure 2()), suggesting a metabolic rather than an osmoadaptive effect in D-glucose challenged fibers. These observations suggest that elevated D-glucose facilitates excitable mechanism(s) that lead to the development of biphasic action potential-induced Ca^2+^ transients in 5-day-old cultured FDB fibers. Note that while control fibers and those challenged with elevated glucose displayed phasic-slow decay AP-induced Ca^2+^ transients, these fibers represented a variable but small fraction of the overall muscle fiber population and were not studied in detail in the present work.

### 3.3. Effect of Electrical Field Stimulation of Alternating Polarity in Fibers with Phasic or Delayed AP-Induced Ca^2+^ Transients

**Figure 3:**
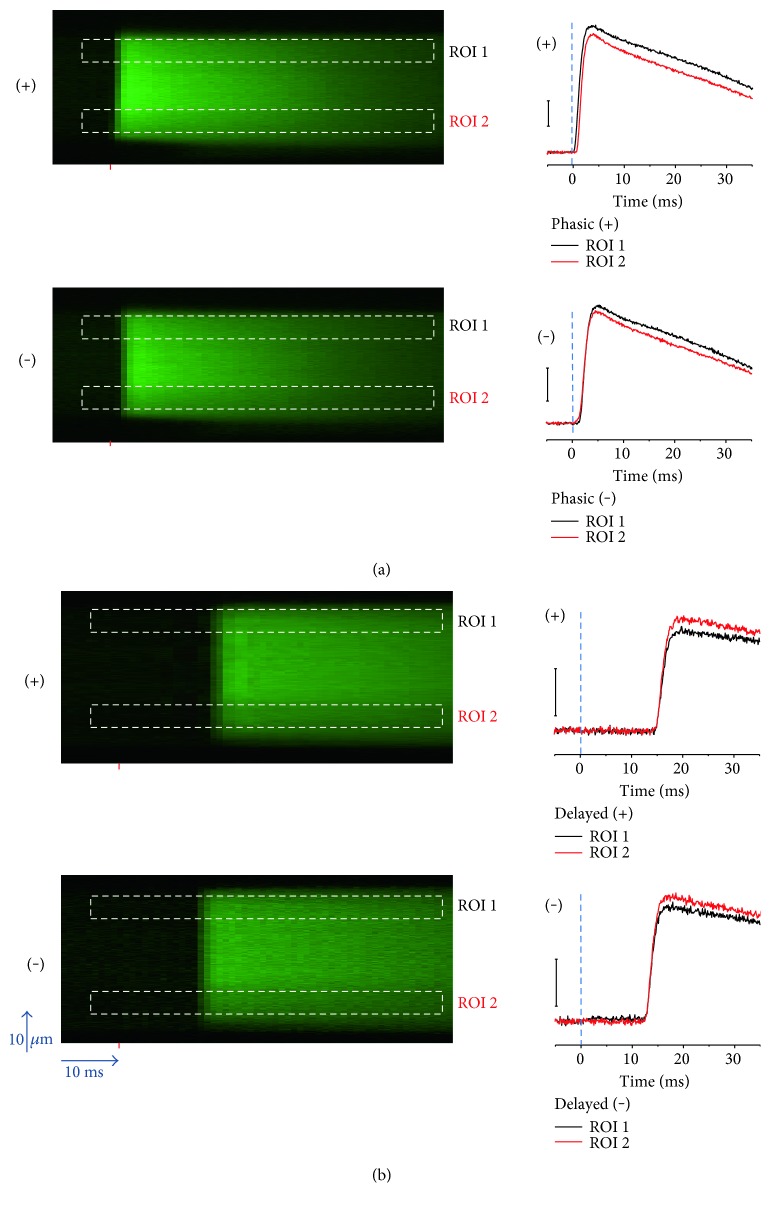
Effect of electrical field stimulation of alternating polarity in fibers with phasic and delayed AP-induced Ca^2+^ transients. (a) *Left*, line-scan images of *phasic* AP-induced Ca^2+^ transients elicited by field stimulation of alternate polarity and measured in two different regions of interest (ROIs); *right*, time course of the phasic AP-induced Ca^2+^ transients measured at two ROIs (ROI 1 and ROI 2) and elicited by the stimulus of positive (*upper traces*) or negative polarity (*lower traces*) at the fiber end where the recordings were made. (b) *Left*, line-scan images of *phasic-delayed* AP-induced Ca^2+^ transients elicited by field stimulation of alternate polarity and monitored in two ROIs; *right*, time course of the delayed AP-induced Ca^2+^ transients measured at two ROIs and elicited by the stimulus of positive (*upper traces*) or negative polarity (*lower traces*) at the fiber end where the recordings were made. The red mark in line-scan images indicates the time when the field stimulus was applied; the dashed rectangles illustrates the location of the ROIs.

To examine the propagation time for AP-induced Ca^2+^ signals, fibers were subjected to suprathreshold field stimulation of alternate polarity. The Ca^2+^ transients in fibers with phasic responses occur with a similar short latency (<2 ms) in response to external stimulation of either polarity (Figure 3(a)), indicating AP-induced Ca^2+^ transients. The time course of the Ca^2+^ transients evaluated at two subsarcolemmal regions on opposite sides of the fiber (ROIs 1 and 2 in Figure 3(a)) reveals that suprathreshold pulses elicited synchronous AP-induced Ca^2+^ transients across the fiber width.

In fibers with phasic-delayed Ca^2+^ transients, the response can occur after a delay (3–15 ms) and could, in principle, result from alterations in AP propagation which translate into AP-induced Ca^2+^ propagation deficits (i.e., slow propagation). We wanted to evaluate whether the delay was caused by the absence of a direct response to stimulation at the site of imaging combined with a slow propagation of an AP from the fiber end away from the recording site and towards the recording site. If this were the case, then inverting the polarity of the stimulus applied to a phasic delayed fiber would trigger an AP-induced Ca^2+^ transient with a considerably shorter delay at the site of recording since propagation would no longer be required.  Figure 3(b) shows AP-induced Ca^2+^ transients in a fiber with delayed responses using pulses of opposite polarity. As in the phasic fiber, suprathreshold pulses elicited synchronous AP-induced Ca^2+^ transients across the fiber width. The comparison of the time course of AP-induced Ca^2+^ transients measured at the same fiber end but with field stimuli of opposite polarity shows similar delayed time courses and only a modest shift (<3 ms) in their latency upon polarity change. This finding suggests that the Ca^2+^ transients are not delayed because of the time for propagation of the action potential or another signal from one end to the other end of the fiber. In that case, the signal recorded at the end of the fiber where the action potential is initiated would not exhibit the delay. However, this was *not* observed. Since the delay was similar for both polarities of stimulation (i.e., with the AP initiated either at the recording site or at the other end of the fiber), we conclude that the delay could be due to either a delay in the activation of the action potential (prior to its propagation along the fiber) or a delay in the activation of the Ca^2+^ transient after the action potential propagation along the fiber when the action potential is activated at the opposite end of the fiber where the recording is taking place. These possibilities are considered further in the discussion.

### 3.4. Blockers of Mechanosensitive Ion Channels, Ca^2+^-Dependent K^+^ Channels, and Na_v_1.5 Channels Do Not Affect Biphasic Action Potential-Induced Ca^2+^ Transients Elicited by a Single Field Stimulus

Next, we investigated whether modifications of the excitable properties of the muscle fiber could account for the occurrence of the biphasic phenotype. We used blockers of ion channels known to modulate the membrane potential and AP properties of the skeletal muscle.

**Figure 4:**
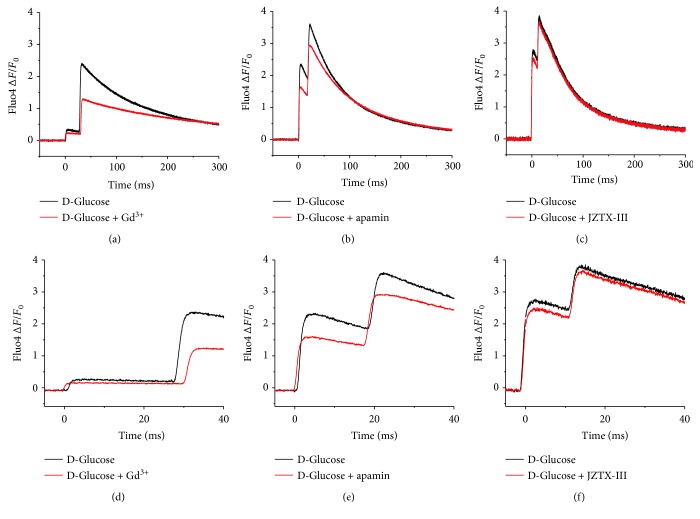
Inhibition of ion channels known to modulate the excitable properties of skeletal muscle: impact on biphasic AP-transients on 5-day old cultured fibers challenged with elevated glucose. Representative time course of a biphasic AP-induced Ca^2+^ transient (left panels) measured in fibers challenged with elevated D-glucose (25 mM; 48 h.) before (black traces) and 10 minutes after (red traces) the treatment with gadolinium (100 *μ*M) (a), apamin (1 *μ*M) (b), and JZTX-III (1 *μ*M) (c). Panels (d–f) are zoomed-in versions of the records shown in (a–c) to better appreciate biphasic responses before and after channel blockers addition. No significant changes in time course of the Ca^2+^ transient (i.e., interspike interval) were found in fibers challenged with 25 mM D-glucose-exposed fibers and treated with gadolinium (*n* = 8 fibers; *p* > 0.05, two-sample paired Student's *t*-test), apamin (*n* = 8 fibers; *p* > 0.05, two-sample paired Student's *t*-test), or JZTX-III (*n* = 6 fibers; *p* > 0.05, two-sample paired Student's *t*-test), when compared to fibers challenged with D-glucose.

The mechanosensitive ion channels (MsC) in the skeletal muscle are activated by membrane stretch and strong membrane depolarization and are permeable to Na^+^ and divalent cations [40]. Increased activity of MsC could cause elevated resting Ca^2+^ levels and/or membrane depolarization [37]. We hypothesized that MsC-induced depolarization would eventually trigger an ectopic AP-induced Ca^2+^ transient. To test whether the occurrence of the biphasic action potential-induced Ca^2+^ transient depended on MsC, the time course of fluo-4 Ca^2+^ elicited by field stimulation and a priori identified as a biphasic signal was measured in control external solution, followed by the addition of gadolinium (Gd^3+^, 100 *μ*M; Figure 4). Ten minutes after the application of Gd^3+^, the Ca^2+^ signal was reassessed. The addition of Gd^3+^ did not affect the time course of the biphasic response. Gd^3+^ did not alter the interspike interval significantly (*n* = 8 fibers; *p* > 0.05, two-sample paired Student's *t*-test); although the amplitude of the Ca^2+^ signal was reduced after the Gd^3+^ addition, this effect was not further evaluated. After washout of Gd^3+^, the amplitude of the Ca^2+^ transient remained reduced (data not shown). The above results suggest that MsC do not contribute to the occurrence of the biphasic action potential-induced Ca^2+^ transients in 5-day-old cultured muscle fibers.

The calcium-sensitive potassium channel with small potassium conductance, K_Ca_2.3, is normally expressed at low level; however, its expression is markedly increased in denervated and myotonic dystrophy muscle [41]. K_Ca_2.3 channel activity in the T-tubules of denervated skeletal muscle causes a local increase in potassium ion concentration that leads to hyperexcitability [38]. Because of their involvement in hyperexcitability, we next considered the possibility that K_Ca_2.3 could be involved in the development of the biphasic action potential-induced Ca^2+^ transient. To test whether the occurrence of the biphasic Ca^2+^ transient involved K_Ca_2.3 channels, the time course of fluo-4 Ca^2+^ elicited by field stimulation was measured in a control external solution, followed by the addition of apamin (1 *μ*M) (Figure 4). K_Ca_2.3 channels can be blocked by apamin [42]. Ten minutes after the application of apamin, the Ca^2+^ signal was measured again. As in the case of MsC, the addition of apamin did not alter the interspike interval significantly (*n* = 8 fibers; *p* > 0.05, two-sample paired Student's *t*-test), although a reduction in the amplitude of the Ca^2+^ signal was also observed (Figure 4). These results suggest that K_Ca_2.3 channels do not contribute to the occurrence of the biphasic action potential-induced Ca^2+^ transients.

The expression of Na_v_1.5 channels is low in 1- to 2-day-old cultured muscle fibers. However, Na_v_1.5 expression increases in fibers cultured for over 3 days [43–45]. This Na_v_1.5 increased expression could explain the occurrence of abnormal excitability and Ca^2+^ signals. To test whether increased Na_v_1.5 function is involved in the altered action potential-induced Ca^2+^ signals seen in 5-day-old cultured fibers, we exposed the fibers to JZTX-III, a Na_v_1.5 channel blocker [39, 46]. The addition of JZTX-III (1 *μ*M; 10 min) to the external solution did not affect the time course of the biphasic response (Figure 4). JZTX-III caused a nonsignificant reduction in the interspike interval (17.3 ± 3.8 ms in D-glucose versus 15.8 ± 3.3 ms in D-glucose treated with JZTX-III, *n* = 6 fibers, 2 mice per group; *p* = 0.509, two-sample paired Student's *t*-test). Contrary to Gd^3+^ or apamin, the addition of JZTX-III to the recording solution did not reduce the amplitude of the AP-induced Ca^2+^ signals (Figure 4). These results suggest that Na_v_1.5 channels do not contribute to the occurrence of the biphasic action potential-induced Ca^2+^ transients.

## 4. Discussion

Numerous studies have investigated how changes in skeletal muscle excitability, Ca^2+^ signaling, and contractility occur in acute and long-term hyperglycemia [12, 13, 20, 21, 47, 48]; however, few studies have examined the impact of diabetes mellitus on the excitability [22], contractility [49], and Ca^2+^ signaling [24] of the skeletal muscle at the cellular level. In particular, little is known about the temporal properties of AP-evoked Ca^2+^ signals during acute hyperglycemia. Using an in cellulo model and high-speed confocal Ca^2+^ imaging, we assessed the impact of acute elevated extracellular glucose (48 h.) on the temporal properties of AP-evoked Ca^2+^ signals. The present study shows that muscle fibers cultured in control medium (5 mM D-glucose) for 5 days display 4 distinct temporal waveforms of AP-induced Ca^2+^ transients: phasic, biphasic, phasic delayed, and phasic-slow decay, in order of predominance. Our study also shows that fibers challenged with elevated extracellular D-glucose (25 mM for 48 h; a condition that could mimic severe uncontrolled hyperglycemia) also exhibit these 4 distinct patterns. However, under these conditions, the biphasic pattern is the predominant waveform, suggesting that elevated glucose promotes the biphasic responses. To our knowledge, this study is the first report of these abnormal AP-induced Ca^2+^ signals in relation to elevated glucose in fibers of normal morphology.

How was a short (1 ms) field stimulus capable of generating such delayed (>3 ms) or biphasic AP-induced Ca^2+^ transients? We have previously shown that the electrode array used in our study allows for the application of field pulses resulting in the depolarization of the end of the fiber close to the cathode, and hyperpolarization of the opposite end of the fiber (near the anode) [36]. In *phasic* fibers, pulses of alternating polarity elicited propagated AP-induced Ca^2+^ transients at the end of the fiber facing the cathode, near the recording site, and its longitudinal propagation along the fiber [36] (see Figure 3()). The time to Ca^2+^ transient peak following single AP stimulation occurs in ~1-2 ms in phasic muscle fibers. The delayed responses (time to Ca^2+^ transient peak > 3 ms) and the second phase of biphasic responses were variable from fiber to fiber. We do not currently know the nature of this variation. We found that fibers with delayed AP-induced Ca^2+^ transient pulses of opposite polarity resulted in subtle latency changes of the Ca^2+^ transient (see Figure 3()). This implies that the delay is *not* due to slow AP propagation through the T-tubule system [50, 51], which would cause a major delay for Ca^2+^ transients initiated at the opposite end from which the recordings are made, but not in responses initiated at the same end where recording occurs.

In the case of *phasic-delayed* fibers, we hypothesize that increased transient outward currents, like K_v_1.4 and K_v_3.4 type-A K^+^ channels, channels expressed in skeletal muscle that oppose membrane depolarization [52, 53], will activate at the cathode near the recording site and will cause membrane potential to reach AP threshold with a delay longer than the stimulus. This delay in AP initiation will cause the observed delay of the AP-induced Ca^2+^ transients in the depolarized end of the fiber near the recording site. The fiber end undergoing hyperpolarization will only be depolarized after the delayed AP is initiated at the other end, followed by rapid propagation of the AP along the fiber. Thus, the (relatively long) delay will be similar at both ends of the fiber for a given polarity stimulation or at the same end of the fiber with alternating polarity stimulation (Figure 2(b)), as observed. Alternatively, AP initiation could have no delay at both ends of the fiber (and/or for both polarities of stimulation), but the Ca^2+^ release response could be delayed due to some as yet undetermined mechanism. In fibers with *biphasic* responses, the negative electrode induces an AP, which triggers the first AP-induced Ca^2+^ transient that propagates towards the positive electrode. The other end, subjected to hyperpolarization, could display voltage sags that counteract hyperpolarization and contribute to rebound membrane potential, triggering the second AP that will propagate toward the other end near to the recording site. Increased inward currents activated by hyperpolarization, such as Kir2.1 channels [54], could explain the membrane potential rebound. Thus, both fiber ends exhibit both a phasic and a delayed response. The Ca^2+^ transients seeing in *phasic-slow decay* fibers could arise from differences in Ca^2+^ binding and transport [55]. These possibilities, and others, require further experimental investigation.

Our results show that the inhibition of MsC with Gd^3+^ did not affect the time course of biphasic AP-induced Ca^2+^ transients during acute hyperglycemia. Note that Gd^3+^ is a nonspecific channel blocker; in addition to inhibiting MsC, it also blocks other ion channels such as voltage-gated K^+^, Na^+^, and L-type Ca^2+^ channels [56]. Fiber treatment with apamin, a K_Ca_ channel blocker [42], did not reverse the effects of elevated glucose on biphasic AP-induced Ca^2+^ transients. Similarly, JZTX-III, a Na_v_1.5 channel blocker [39, 46], did not alter the occurrence of the biphasic responses in fibers challenged with elevated glucose. These findings suggest that neither MsCs, K_Ca_, nor Na_v_1.5 plays a role in the origin of the biphasic AP-induced Ca^2+^ transients. It is yet to be determined whether other ion channels play a role in the abnormal AP-induced Ca^2+^ transients in long-term cultures and/or exposure to elevated glucose.

Ca^2+^ signals are essential in numerous aspects of muscle function [57, 58]. A previous study reported the occurrence of local Ca^2+^ signals by acute (≤1 h.) hyperosmotic stress in the cell periphery of cultured muscle fibers [59]. Whether local Ca^2+^ signals are present in muscle fibers challenged with hyperosmotic stress induced by elevated glucose for more prolonged periods (>24 h.) remains to be determined.

Do these defects on AP-induced Ca^2+^ signals seen in long-term cultured fibers and experimental hyperglycemia occur in patients with diabetes? Most adults with diabetes have at least one coexisting condition, either acute or chronic [60]. Muscle weakness and fatigue are common complaints of diabetic patients during periods of acute [13] and long-term hyperglycemia [61] and are also common in muscle disuse atrophy [62, 63]. Because Ca^2+^ signals and excitable properties are critical for skeletal muscle function [52, 57], we hypothesize that if the changes in excitability and abnormal Ca^2+^ signals observed in five-day-old cultured fibers occur in vivo, these could contribute to the development of muscle weakness, fatigue, and diabetic myopathy. Further work exploring the underlying mechanisms and relationship between diabetes and skeletal muscle disuse (denervation/physical inactivity, etc.) would be of pathophysiological interest.

In this study, we used an in cellulo model of hyperglycemia using 25 mM glucose for 1-2 days. This paradigm is an extreme model of hyperglycemia, and it is restricted to a short spectrum of metabolic abnormalities and hormonal changes seen in diabetes (i.e., severe uncontrolled diabetes). The abnormalities in AP-induced Ca^2+^ signals that we observed may be influenced or caused by fiber disuse and/or denervation which may occur in long-term cultured muscle fibers [25, 64] and in some extent by in vitro dedifferentiation [33]. Both muscle disuse/denervation and dedifferentiation are characterized by abnormal excitability [33, 45]. Because ara-C treatments minimize the dedifferentiation process [25, 33] (see also Supplementary Figure 2), we believe that dedifferentiation could play a minor role in our observations. Nevertheless, the cultured muscle fibers used here represent a cellular model of muscle disuse/denervation [25, 65] and is a valuable alternative to animal studies to explore severe and acute effects of hyperglycemia on the function of skeletal muscle fibers.

## Supplementary Material

Supplemental Figure 1. Acute (1 h.) exposure to elevated D-glucose enhances AP-evoked Ca2+ transients in cultured muscle fibers. Comparison of the time course of AP-induced Ca2+ transients in muscle fibers before (black trace; control medium) and after 1 h. challenge with 25 mM D-glucose, cyan trace (A), or after 1 h. challenge with 25 mM L-glucose, red trace (B). Summary of rest and at peak indo-1 ratio measurements for fibers exposed to 25 mM elevated D- or L-glucose (C). No significant (N.S) changes in resting indo-1 ratio were found in fibers challenged with either 25 mM elevated D- or -L-glucose. Exposure for 1 h. with D-glucose induced a significant increase in peak indo-1 ratio. ∗Indicates p < 0.05 compared with control, two-sample unpaired Student's t-test, n = 14-15 fibers, 3 mice.Supplemental Figure 2. Ara-C treatments minimize in vitro de-differentiation. Exemplar transmitted light images of muscle fibers cultured in serum-containing medium (A) and serum-containing medium and treated with cytosine β-D-arabinofuranoside (B; ara-C, 10 µM; 24 h.) to minimize de-differentiation. Images of the same fibers were taken at day 1 (upper rows) and day 5 (lower rows) after fiber plating. Scale bar 100 µm. Note that in fibers treated with ara-C, the morphology was retained after 5 day in culture, whereas cell proliferation, intercellular fusion, and fiber sprouting were evident in the serum-containing medium
